# Longitudinal consistency of source-space spectral power and functional connectivity using different magnetoencephalography recording systems

**DOI:** 10.1038/s41598-021-95363-2

**Published:** 2021-08-11

**Authors:** Lennard I. Boon, Prejaas Tewarie, Henk W. Berendse, Cornelis J. Stam, Arjan Hillebrand

**Affiliations:** 1 Department of Neurology, Amsterdam Neuroscience, Amsterdam UMC, Vrije Universiteit Amsterdam, De Boelelaan 1117, 1081 HV Amsterdam, The Netherlands; 2 Department of Clinical Neurophysiology and Magnetoencephalography Center, Amsterdam Neuroscience, Amsterdam UMC, Vrije Universiteit Amsterdam, De Boelelaan 1117, 1081 HV Amsterdam, The Netherlands

**Keywords:** Neuroscience, Medical research, Neurology

## Abstract

Longitudinal analyses of magnetoencephalography (MEG) data are essential for a full understanding of the pathophysiology of brain diseases and the development of brain activity over time. However, time-dependent factors, such as the recording environment and the type of MEG recording system may affect such longitudinal analyses. We hypothesized that, using source-space analysis, hardware and software differences between two recordings systems may be overcome, with the aim of finding consistent neurophysiological results. We studied eight healthy subjects who underwent three consecutive MEG recordings over 7 years, using two different MEG recordings systems; a 151-channel VSM-CTF system for the first two time points and a 306-channel Elekta Vectorview system for the third time point. We assessed the within (longitudinal) and between-subject (cross-sectional) consistency of power spectra and functional connectivity matrices. Consistency of within-subject spectral power and functional connectivity matrices was good and was not significantly different when using different MEG recording systems as compared to using the same system. Importantly, we confirmed that within-subject consistency values were higher than between-subject values. We demonstrated consistent neurophysiological findings in healthy subjects over a time span of seven years, despite using data recorded on different MEG systems and different implementations of the analysis pipeline.

## Introduction

Magnetoencephalography (MEG) allows for measurement of fluctuating magnetic fields induced by neuronal currents. It provides information about normal and pathological brain activity with excellent temporal and good spatial resolution^1,2^. Communication between distributed brain regions is assumed to be reflected in the statistical relationships between the regions’ time series of activity, referred to as functional connectivity^3^. Disruption of resting-state functional interactions between brain regions is considered to be a final common pathway in many brain disorders^4,5^.

Longitudinal MEG studies are essential for a full understanding of disease-specific pathophysiological mechanisms and the development of (changes in) functional brain networks over time, but can be complicated by changes in site-specific factors, such as environmental noise and the MEG recording system itself. In our center, a VSM-CTF system was replaced by an Elekta Vectorview system, resulting in changes in both hardware and software, including a different number of sensors (151 third-order axial gradiometers versus 306 magnetometers/planar gradiometers, respectively), and a different beamformer implementation. Although this has hardly been studied, the type of MEG sensor that is used affects the MEG signals that are recorded: For a given number of measurement channels, axial magnetometers provided more information than vector magnetometers^6^. In addition, it has been shown^7^ that radial gradiometers (i) have a better signal-to-noise ratio than radial magnetometers, (ii) have slower signal-strength decay with increasing depth than planar gradiometers, and (iii) of the third order are less sensitive to head-motion and vibrational noise than those of the first order.

Since replacement of a recording system is a common and reoccurring event at many centers, we considered it important to evaluate the consistency of longitudinal MEG data obtained in a group of healthy volunteers using different MEG recording systems. We longitudinally analyzed data from eight healthy volunteers who all underwent a resting-state MEG recording at three time points as controls in a longitudinal cohort study: baseline (BL); follow-up 4 years after baseline (FU1); follow-up 7 years after baseline (FU2). BL and FU1 were recorded using a VSM-CTF MEG system, FU2 using an Elekta Vectorview system.

We applied an often-used atlas-based beamforming approach^8–11^ to project MEG signals into source space, allowing for interpretation in an anatomical context^12,13^ and comparison across recording sessions and systems. We assessed consistency of within-subject spectral power and functional connectivity over time and across different MEG systems and compared this with between-subject findings. We hypothesized that the within-subject consistency between different MEG systems would be (i) comparable to the within-subject consistency of data recorded using the same MEG system; (ii) higher than the between-subject consistency.

## Materials and methods

### Participants

Data from healthy participants were recorded in the context of a longitudinal case-control follow-up study in Parkinson’s disease^14–18^ over a period of 7 years (the first follow-up visit being ~ 4 years after inclusion). The healthy participants did not suffer from neurological or psychiatric diseases and did not use any drugs or medication. From an initial group of 20 healthy participants, 10 had undergone an MEG recording at all three time points. Two subjects were excluded from further analysis due to an excess of head movement during the baseline MEG recordings. Hence, data from 8 healthy participants aged 45–72 years (55 ± 5.92) were used. The study was approved by the Medical Ethics Committee of Amsterdam UMC, location VU University Medical Center (Amsterdam, The Netherlands), and all participants gave written informed consent before participation. All methods were carried out in accordance with relevant guidelines and regulations.

### Data acquisition

BL and FU1 MEG data were acquired using a 151-channel whole-head MEG system with axial gradiometers (CTF systems Inc., Vancouver, Canada) in an eyes-closed resting-state condition for 5 min while subjects were seated inside a magnetically shielded room. A recording passband of 0.25–200 Hz, and sample rates of 312.5 (BL) and 625 Hz (FU1) were used, and a 3rd order software gradient was applied^19^. At the beginning and end of each recording the head position relative to the coordinate system of the helmet was assessed by leading small currents through three head position indicator (HPI) coils attached to the left and right pre-auricular points and the nasion.

FU2 MEG data were recorded using a 306-channel Vectorview system with 102 magnetometers and 204 planar gradiometers (Elekta Neuromag, Oy, Helsinki, Finland) in an eyes-closed resting-state condition for 5 min in a supine position. An online anti-aliasing (410 Hz) and high-pass filter (0.1 Hz) were used and the sample rate was 1250 Hz. The head position relative to the MEG sensors was recorded continuously using the signals from four HPI coils. The coil positions were digitized before each recording, as well as the outline of the patient’s scalp (~ 500 points), using a 3D digitizer (Fastrak, Polhemus, Colchester, VT, USA).

Structural T1-weighted MR imaging was performed at all three time points (BL; 1.0 T, Impact, Siemens, Erlangen, Germany; FU1 and FU2; 3.0 T, GE Signa HDxt, Milwaukee, WI, USA). In preparation of the MR imaging at BL and FU1, vitamin E capsules were placed at the same anatomical landmarks where the three HPI coils had been placed during MEG-registration.

### Data pre-processing

**Figure 1:**
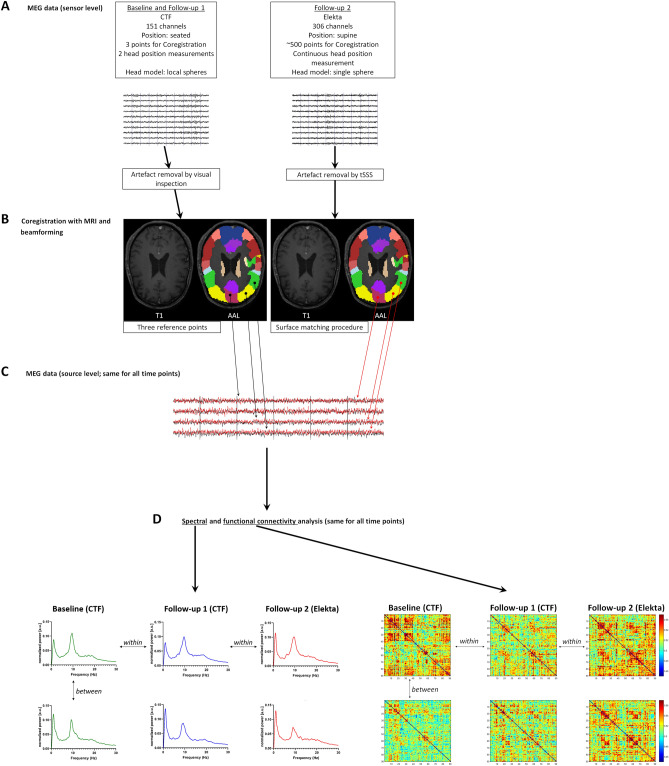
Schematic overview of preprocessing and analysis. Five minutes of eyes-closed, resting-state MEG recordings (**A**) took place on a VSM-CTF system (baseline (BL; t = 0) and follow-up 1 (FU1; t = 4 years), and on an Elekta Vectorview system (FU2; t = 7 years). (**B**) Data from all channels were projected onto an anatomical framework of 90 brain regions (automated anatomical labeling (AAL) atlas), leading to source-space MEG data for all time points (**C**). (**D**) Whole-brain spectral analysis, as well as functional connectivity between all pairs of brain regions, was assessed by means of the leakage-corrected amplitude envelope correlate (AEC). Power spectra and connectivity matrices were compared over time within patients (horizontal arrows), as well as cross-sectionally between patients (vertical arrows).

A schematic representation of the pre-processing steps can be found in Fig. 1A–C.

We used two different pipelines to pre-process the data, each one dedicated to a given hardware. Both FU1 and FU2 MEG-data were downsampled to 312.5 Hz, to obtain the same sample rate as BL. MEG channels that were malfunctioning, for example due to excessive noise, were removed after visual inspection of the data (all by the same observer KTEOD; mean number of excluded channels: BL/FU1 2.4, range 2–7; FU2 6, range 2–11). BL and FU1 data were split into epochs (13.11 s, 4096 samples) and epochs containing strong artefacts were discarded (BL mean 2.1 (range 0–4); FU1 mean 4.6 (range 0–13); KTEOD). In addition, the temporal extension of Signal Space Separation (tSSS) in MaxFilter software (Elekta Neuromag Oy, version 2.2.15) was applied to FU2 data to remove artefacts^20,21^ with a sliding window of 10 s and a subspace correlation-limit of 0.9 (the correlation limit provides a trade-off between removal of noise and preservation of brain signal^22^, where a value of 0.9 was found to be optimal in our specific (urban) environment). Note that tSSS (also) reconstructs the data for the identified malfunctioning channels. Participants’ MEG data were co-registered to their structural MRIs, in case of BL/FU1 through identification of the same anatomical landmarks (left and right pre-auricular points and nasion; estimated co-registration error < 6 mm) in both modalities, and in case of FU2 through a surface-matching procedure, with an estimated resulting accuracy of 4 mm^23^. For all three time points, the automated anatomical labelling (AAL) atlas was used to label the voxels in 78 cortical and 12 subcortical regions of interest (ROIs) in a subject’s co-registered MRI^24,25^. In order to obtain a single time series for a ROI we used each ROI’s centroid as representative for that ROI^9^. A scalar beamformer^12,26^ was used to reconstruct beamformer weights for each centroid using broad-band data (0.5–48 Hz; mean 201 s (range 131–262 s) for BL, mean 238 s (range 197–288 s) for FU1, and mean 297 s (range 288–341 s) for FU2), and using Synthetic Aperture Magnetometry^27^ for BL and FU1, and Elekta’s beamformer implementation (version 2.2.15; Elekta Neuromag Oy), which finds the optimum beamformer orientation through an eigendecomposition^28^, for FU2. The beamformer used lead fields for equivalent current dipoles at the centroid locations, using a multi-sphere^29^ or single-sphere head model for BL and FU1 or FU2, respectively. Broad-band data (0.5–48 Hz) were subsequently projected through the normalized^30^ beamformer weights in order to project the sensor signals to source space, i.e. broadband (0.5–48 Hz) time-series of neuronal activity were reconstructed for each centroid of the 90 ROIs.

Subsequently, FU2 time-series were downsampled (4×) and split into epochs of 4096 samples (13.11 s) as well. All beamformed data was used for further analysis (BL range 10–20 epochs, FU1 15–18 epochs and FU2 19–22 epochs), hence no further epoch-selection took place.

### Data analysis

Spectral power and functional connectivity analyses were performed using in-house software (BrainWave, version 0.9.152.12.26; CJS, available from http://home.kpn.nl/stam7883/brainwave.html). For each subject and time point separately, we estimated the overall spectral power (0.5–48 Hz) averaged over all ROIs and epochs, normalized based on the area under the curve. Peak frequency values were determined within the 4–13 Hz frequency range. In addition, for each epoch, we band-pass filtered the data into the alpha (8–13 Hz) and beta (13–30 Hz) band using a Fast Fourier transform to produce a so-called ‘brickwall’ filter. We subsequently corrected for signal leakage by pairwise orthogonalisation (in both directions^31^), and estimated functional connectivity by amplitude envelope correlation (AEC), separately for each epoch. The AEC computes the correlation between the amplitude of the envelopes of two time series obtained with the Hilbert transform (in our case) or wavelet analysis^31–33^. To adjust for any negative correlations, 1 was added to the raw AEC values and subsequently divided by 2. The AEC was calculated for all possible pairs of ROIs, leading to a 90 × 90 weighted adjacency matrix for each epoch. For each subject, the matrices were then averaged over epochs. A schematic representation of the spectral power and functional connectivity analyses can be found in Fig. 1D.

### Statistical analysis

First, measures of within- (longitudinal) and between-subject (cross-sectional) consistency in spectral power and functional connectivity were calculated for each individual. Within-subject correlations were assessed between BL–FU1, FU1–FU2, and BL–FU2. Between-subject consistency was calculated by taking the average of the cross-sectional consistency of one subject with the other subjects. The two sample Kolmogorov–Smirnov test was used to estimate differences in the shape of power spectra (as a measure of consistency) within- (longitudinal) and between (cross-sectional) subjects. To study consistency in functional connectivity within- and between subjects, we compared functional connectivity matrices, averaged over all epochs of each individual. We vectorized the average matrix (while excluding the diagonal) and calculated Spearman correlations between these vectors, since the functional connectivity data were not Gaussian distributed.

Second, we tested the hypothesis that no significant differences between within-subject correlations were present across time, and hence that two recordings performed on the same MEG system (BL–FU1) would show comparable results with recordings performed on two different MEG systems (FU1–FU2 and BL–FU2). We also tested the hypothesis that no significant differences in between-subject correlations were present over time, in which a p-value > 0.05 suggests there is no difference. As all values conformed to the normality assumption, these correlations were tested using repeated measures ANOVAs.

Third, we tested the hypothesis that within-subject correlations were higher than between-subject correlations. We averaged the within-subject correlations (assessed between BL–FU1, FU1–FU2, and BL–FU2), and compared this with the average between-subject correlations (BL, FU1, and FU2) using two-tailed unpaired t-tests.

## Results

### Spectral power

**Figure 2:**
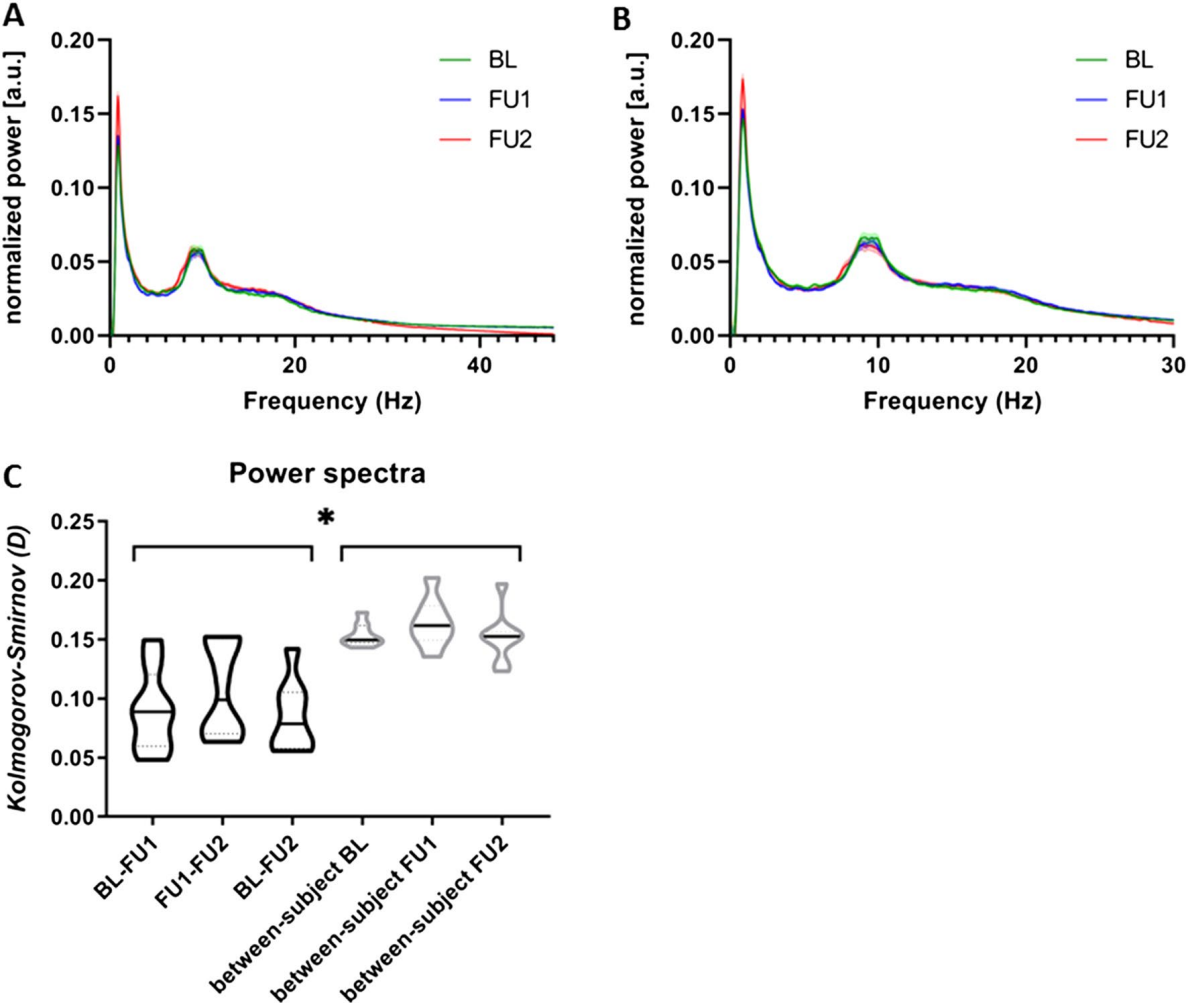
Power spectral analysis. Whole-group normalized power spectra (± standard error of the mean) at three time points, averaged over all brain regions, between 0.5 and 48 Hz (**A**) and between 0.5 and 30 Hz (**B**). (**C**) Violin plots summarizing the within (black) and between-subject (grey) test statistics (unit: D) between power spectra (0.5–30 Hz) at different time points using the two-sample Kolmogorov–Smirnov test. Higher values represent a larger difference in the shape of the power spectra. No significant differences between average within-subject values nor between average between-subject values were found using a repeated measures ANOVA (Table 1). Note that on average, the within-subject comparability was statistically significantly higher (as the D values were lower) than the between-subject comparability (*t*(46) = 7.77; *p* < 0.001; average values also provided in Table 1). *p < 0.05.

Overall normalized spectral power averaged over all subjects is shown in Fig. 2A,B. Figure 2A demonstrates broadband spectral power (0.5–48 Hz). As can be observed from this figure, the normalized power spectra from the three time points overlapped in the range of 0.5–30 Hz, but the spectra obtained from BL and FU1 (CTF data) showed more gamma power (30–48 Hz) than the power spectrum from FU2 (Elekta data). As the presence of artefacts may have contributed to this difference [for example magnetometers/planar gradiometers of the Elekta Vectorview system (FU2) may be less sensitive to muscle artefacts or these artefacts may be suppressed more optimally by using tSSS compared to the use of synthetic third-order gradiometers in the CTF system (BL, FU1)], we subsequently restricted the spectral analysis to 0.5–30 Hz (Fig. 2B).

We estimated the within- and between-subject difference in shape (as a measure of consistency) of the power spectra using the two sample Kolmogorov–Smirnov tests (Fig. 2C), in which lower values represent higher consistency. The average within- and between-subject consistency did not differ significantly between time-points (Table 1). Importantly, comparison of power spectra within subjects over time showed significantly higher consistency than power spectra between subjects (cross-sectional; Fig. 2C). In addition, the peak frequency did not significantly differ between time points: BL (mean 8.78 SD 0.87), FU1 (mean 8.78 SD 0.92), FU2 (mean 8.91 SD 0.89), (F(2, 7) = 0.76, *p* = 0.53, ηp^2^ = 0.24).

**Table 1:** Within- and between-subject consistency values, expressed as mean (standard deviation). Note that in case of spectral power, lower Kolmogorov–Smirnov 2 test statistic values represent a higher consistency. In case of the functional connectivity analyses (leakage corrected AEC), higher Spearman’s rho values indicate a higher consistency.

	Within-subject			Statistics	Between-subject			Statistics
	BL–FU1	FU1–FU2	BL–FU2		BL	FU1	FU2	
Spectral power*	0.090 (0.035)	0.107 (0.040)	0.085 (0.030)	F(2, 7) = 1.151, ηp^2^ = 0.277, *p* = 0.378	0.154 (0.010)	0.164 (0.021)	0.152 (0.022)	F(2, 21) = 1.031, ηp^2^ = 0.001, *p* = 0.374
Alpha cAEC	0.380 (0.167)	0.407 (0.156)	0.438 (0.095)	F(2, 7) = 0.405, ηp^2^ = 0.021, *p* = 0.684	0.279 (0.063)	0.211 (0.072)	0.317 (0.068)	F(2, 21) = 5.028, ηp^2^ = 0.046*, p* = 0.016**
Beta cAEC	0.440 (0.121)	0.453 (0.099)	0.426 (0.098)	F(2, 7) = 2.26, ηp^2^ = 0.430, *p* = 0.185	0.319 (0.059)	0.338 (0.040)	0.371 (0.051)	F(2, 21) = 2.132, ηp^2^ = 0.011, *p* = 0.144

*BL* baseline, *FU1* follow-up 1, *FU2* follow-up 2, *ηp*^*2*^ partiel eta squared (effect size); Statistics on correlation values over time was performed using repeated measures ANOVAs.

*Consistency values expressed as Kolmogorov–Smirnov 2 test statistic.

**Post-hoc paired *t*-tests; BL–FU1 *t*(7) = 2.53, *p* = 0.039; FU1–FU2 *t*(7) = 4.04, *p* = 0.005; BL–FU2 *t*(7) = 1.51, *p* = 0.175.

### Functional connectivity

**Figure 3:**
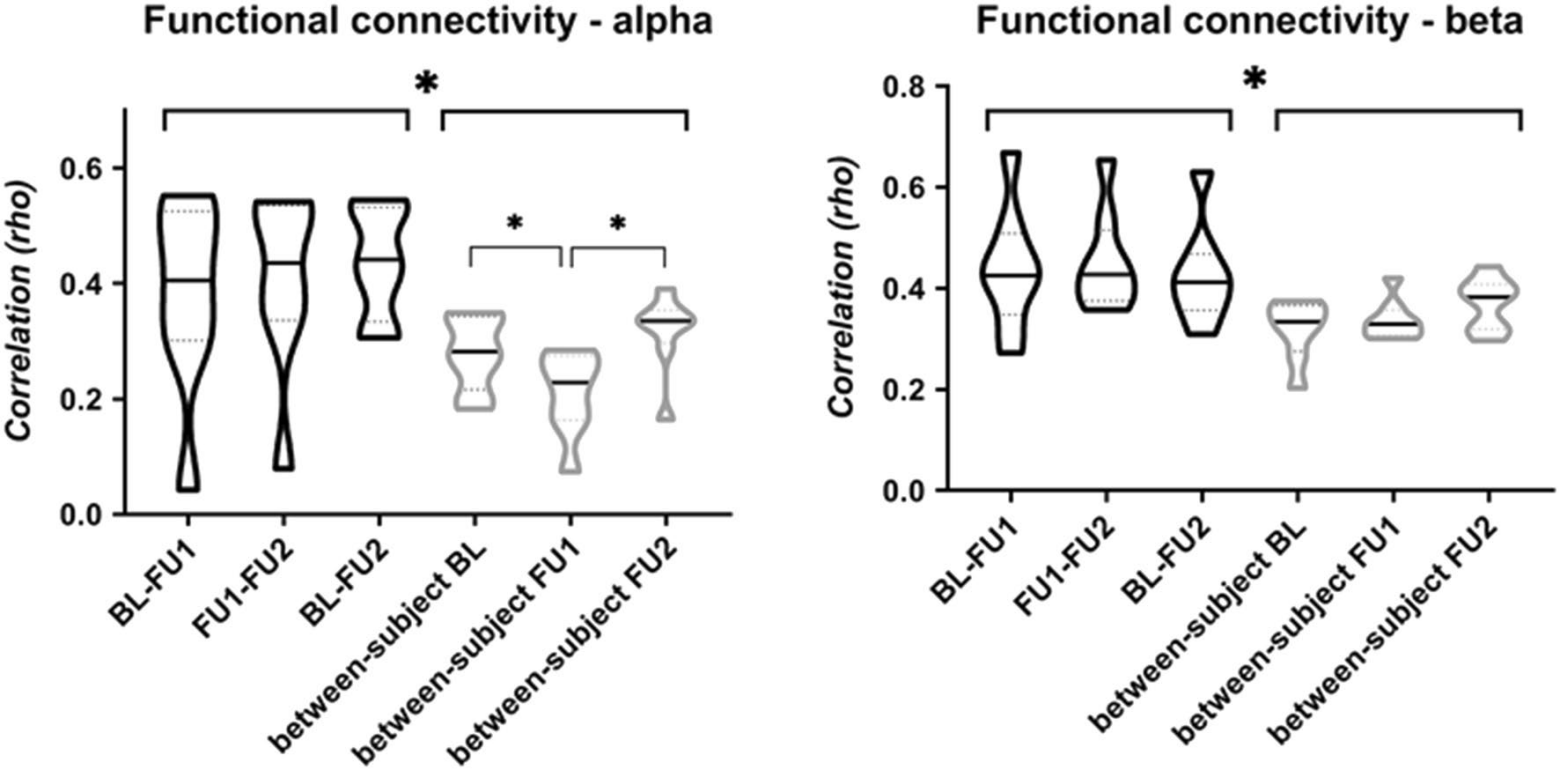
Functional connectivity analysis. Violin plots summarizing within and between-subject correlation values (Spearman’s rho) between functional connectivity matrices at different time points. Connectivity matrices consisted of alpha (8–13) and beta (13–30) band amplitude envelope correlation (AEC) values, corrected for signal leakage. No significant differences were found between average within-subject values using repeated measures ANOVAs, but between-subject alpha band functional connectivity values differed between time points BL–FU1 and FU1–FU2 (Table 1). Note that on average, the within-subject correlations were significantly higher than the between-subject correlations (alpha band t(46) = 4.27, *p* = 0.011; beta band t(46) = 4.12, *p* < 0.001); average values also provided in Table 1). *p < 0.05.

**Figure 4:**
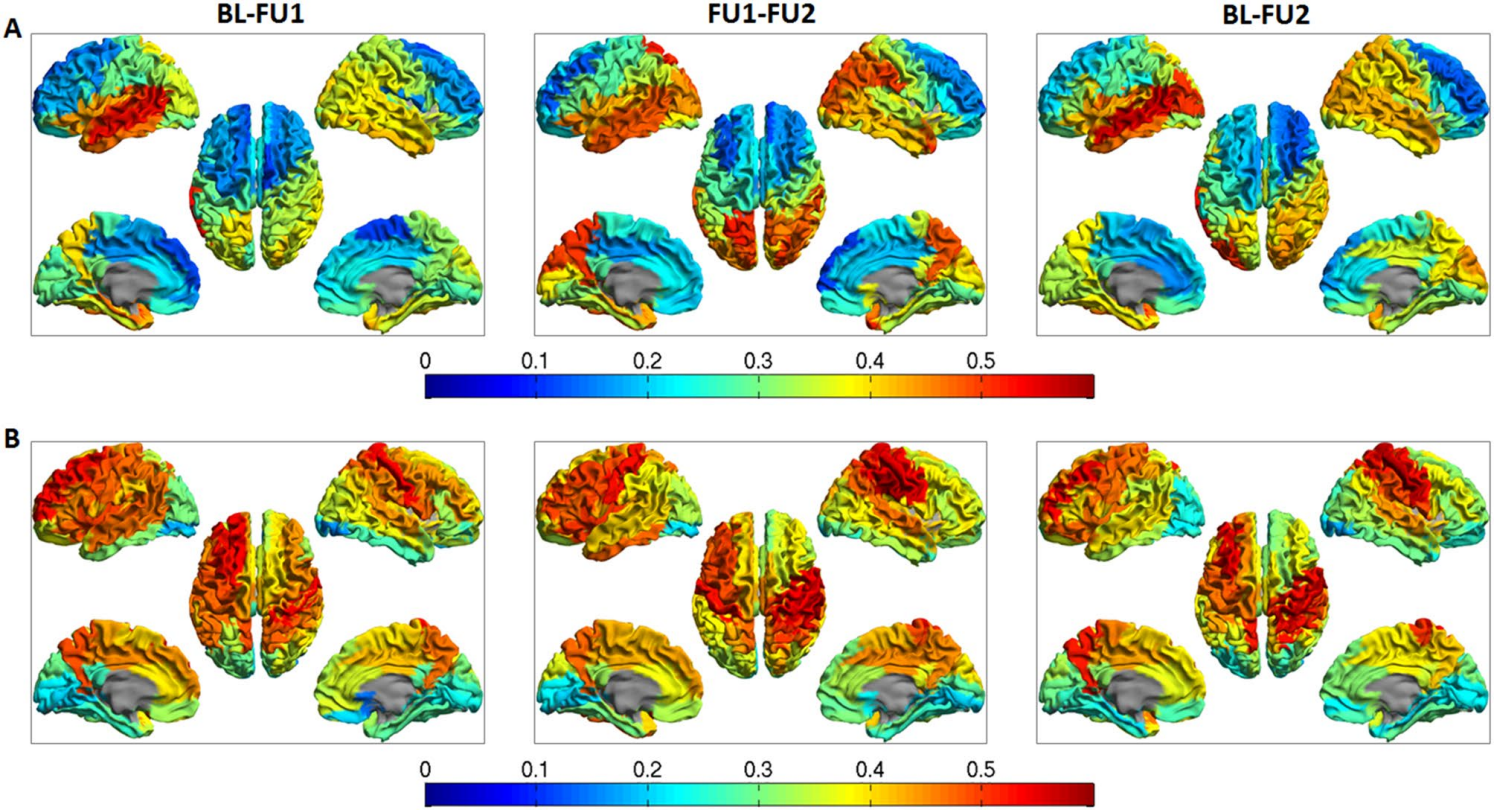
Functional connectivity analysis; region of interest-specific display. Distribution of within-subject functional connectivity correlation values displayed on a parcellated template brain viewed from, in clockwise order, the left, right, left midline, and right midline. For alpha band functional connectivity, the correlation values were highest in the parieto-occipito-temporal cortical brain regions (**A**). For beta band functional connectivity, the correlation values were highest for the central and frontal brain regions (**B**). The subcortical regions had an intermediate level of correlation, both for the alpha and beta band functional connectivity (not shown).

We calculated within- and between-subject correlations between connectivity matrices for the three time points (Fig. 3). For both alpha and beta band functional connectivity, the highest within-subject correlations were found in the correlation with FU2 (BL–FU2 for alpha band FC, FU1–FU2 for beta band FC; in which FU2 represented a different MEG recording system). For alpha band FC, the within-subject correlation values were highest in the parieto-occipito-temporal cortical brain regions (Fig. 4; panel A). For beta band FC, the correlation values were highest for the central and frontal brain regions (Fig. 4; panel B). The average within- and between-subject correlations were not significantly different over time (Table 1). Importantly, both in the alpha and beta band, within-subject correlations were significantly higher than between-subject correlations (Fig. 3).

## Discussion

In this study we have demonstrated that longitudinal within-subject neurophysiological results in healthy subjects were consistent across MEG systems. We demonstrated this by restricting the normalized power spectral analysis to 0.5-30 Hz and using amplitude-envelope coupling as a measure of functional connectivity. Furthermore, within-subject consistency values were significantly higher than between-subject consistency values, confirming that over time, neurophysiological information of individuals is retained.

We here demonstrate the feasibility of a *longitudinal* MEG analysis using data from the same subjects recorded on different MEG systems over a timespan of 7 years. A previous *cross-sectional* study used a median-nerve stimulation paradigm and demonstrated the stability of the location of the N20m response, both in terms of location and latency, within the same subject on three different MEG systems (including a Neuromag Vectorview and CTF-VSM system), although the magnitude of later evoked components was more variable^34,35^. This suggests that cross-sectional pooling of MEG data recorded at different sites may be possible as well. However, other site-specific factors than the MEG system and preprocessing pipeline, such as environmental noise and the site-specific agreement on epoch selection criteria, may complicate such an effort. Future studies should further explore the feasibility of pooling MEG data from different sites (see for example the MRC/EPSRC partnership initiative for MEG in the UK, e.g. Ref.^36^).

Although we reported consistency of spectral power and functional connectivity estimates over time, several factors and sources of error could have negatively affected this, most of which were related to hardware differences between the CTF and Elekta systems. (i) Different number and type of sensors (151 vs 306; axial vs planar gradiometers; third-order gradiometers versus magnetometers and first-order gradiometers). Although the information content of the raw data was therefore different, we performed our analysis using the same number of (virtual) sensors through the use of the same anatomical atlas for all datasets. (ii) The artefact correction method, which was performed manually in case of the CTF system (i.e. rejection of bad data segments) and was done using tSSS in case of the Elekta system (automatic removal of artefacts from the data). This difference may have caused the lower normalized gamma power found at FU2, since tSSS may have removed (e.g. muscle) artefacts from the data more successfully than a visual analysis. In the visual analysis on BL and FU1 data, only data segments with clear artefacts were removed, and less obvious (muscle) artefacts may therefore have been left in the remaining data. Importantly, after excluding the gamma band from the spectral analysis, the power spectra were similar over time/systems. However, the influence of tSSS on gamma activity may not have been the sole explanation for this difference: tSSS may also affect alpha and high-beta relative power, as demonstrated in Supplementary Fig. 2. Another factor of influence may be a different sensitivity of magnetometers/planar gradiometers of the Elekta Vectorview system (FU2) to muscle artefacts. In addition, the use of a sliding window (of 10 s) may introduce artificial discontinuities in the data, although we did not encounter these. (iii) Measurement of head position during the recording (at two time points vs continuous; where the first could lead to an underestimation of actual head movement during recordings). (iv) Co-registration accuracy (three fiducials for co-registration vs surface-matching; the first method being more prone to errors^37^). This may have affected both the spectral analysis and the functional connectivity assessment^38^, although the influence of differences in the co-registration approach was probably limited, as the spatial resolution of the beamforming approach was already reduced through the use of (the centroids of atlas-based) regions-of-interest. (v) Differences in the head model (local spheres vs single sphere) used for the computation of the lead fields. (vi) The implementation of the scalar beamformer differed between the CTF and Elekta systems. (vii) The CTF recordings (BL and FU1) were performed in seated position, whereas the Elekta recordings were performed in supine position. Although not systematically tested, participants may have shown more movement during recordings in the seated position, for example due to subsiding as the recording progresses. On the other hand, participants recorded in the supine position may be more prone to drowsiness. However, this was probably not an issue in our study, as drowsiness is related to a slowing of the dominant background rhythm which we did not find (peak frequency and spectra were consistent over time/systems, despite the fact that we did not select data on the absence of drowsiness). Importantly, in spite of the differences in hardware and the processing pipeline there was no difference in within-subject consistency between BL–FU1 and FU1–FU2. This would seem to indicate robustness of the estimation of spectral power and functional connectivity across recording systems by performing the analyses in source-space.

The present study has some limitations. Firstly, the data collection in this study was not designed with the idea to answer the question whether data recorded on two different MEG system would show comparable results. To better answer this question, the data collection should have included a cross-sectional comparison between two different MEG systems (instead of only a longitudinal comparison between systems). However, as we assume that the adult healthy controls in our analysis have stable brain activity over time (we did not observe a trend over time, nor did another study^39^; moreover in the presence of any significant aging effects our analyses would not have shown neurophysiological results to be stable over time), we believe that we were able to answer this question successfully.

Secondly, we used a leakage-corrected metric of functional connectivity that has previously been demonstrated to be a metric with a good within-subject reproducibility in the alpha band^40^ and to be stable during healthy ageing^39^. As one of the aims of this study was to study its consistency over time (and systems), we did not perform an exhaustive test of available connectivity measures, such as metrics for phase-based coupling^41^, generalized synchronization^42^, or information based metrics^43^. However, both our within- and between-subject alpha band functional connectivity reproducibility values did not reach the levels as reported by Colclough and colleagues (average within-subject correlation ~ 0.55 and average between-subject correlation ~ 0.45)^40^. A possible explanation for the lower within-subject consistency found in our study might be the longer duration between recordings (several years versus several hours).

Thirdly, in contrast to earlier work from our group^14,16,44,45^, no epoch selection took place before our analysis. Epoch selection is generally performed to prevent the inclusion of data segments during which the subject was drowsy and/or which contained artefacts. However, as a previous study demonstrated that high between-session repeatability can be reached by using large amounts of MEG data (> ~ 100 s)^46^, we chose not to perform epoch selection here. As a result, we obtained rather high within-subject reproducibility both regarding spectral power and functional connectivity. Intuitively, without epoch selection, MEG data may contain drowsiness in the recordings, which may affect spectral power. We do however think this effect was limited in this analysis as (i) the reproducibility values of the spectral data were rather good, (ii) the peak frequencies hardly changed between unselected (all) and selected data (n = 5 epochs; based on a previous epoch selection^14,17^): BL *all data*; mean 8.78 Hz, *selected data*; mean 8.75 Hz. FU1 *all data*; mean 8.78 Hz, *selected data*; mean 8.92 Hz FU2 *all data*; mean 8.91, *selected data*; mean 8.92 Hz.

Fourthly, the number of subjects in this study was small. This may have lowered the power to find statistical differences between consistency values over time points (within the same scanner type versus between different scanner types). We are well aware that the absence of statistically significant differences does not mean that our neurophysiological results were the same over time points^47^. However, the observation that the power spectra (0.5–30 Hz) visually aligned, both at the group level and at the individual level (Supplementary Fig. 1), suggests stability.

Fifthly, a disadvantage of the AAL atlas is that some central, frontal and temporal regions span a relatively large surface. Consequently, the centroid (most central voxel) may be relatively distant from the voxel that is most representative for the activity of that brain region. However, in a previous study from our group, we compared the centroid-voxel and peak-voxel approaches (maximum power) and there were no major differences between the two methods for the neurophysiological measures that were assessed^9^. Also, we think we can draw meaningful conclusions on the spatial distribution of the correlations in our analysis, as high FC correlations values were present in central, frontal (beta band FC) and temporal (alpha band FC) brain regions. Interestingly, the highest correlation values are reflected by the posterior cortical alpha rhythm (alpha band FC) and mu rhythm (beta band FC; Fig. 4), which is possibly related to higher signal-to-noise ratio’s in these brain regions. Another reason not to use an atlas with a much denser parcellation scheme is the fact that the number of brain regions of the AAL atlas roughly matches the number of (data-driven) parcels that can be obtained in beamformed resting-state MEG data^48^, indicating that, on average, the resolution of the AAL atlas matches that of beamformed resting-state data. We do however think that optimization of the parcellation approach warrants a separate study, in which existing atlases with different parcellation densities and adaptive parcellation strategies are compared^48^.

Lastly, we draw the conclusion that the estimation of spectral power and functional connectivity is robust across recording systems, but this conclusion applies to the specific analysis pipeline that we used. This includes the fact that we had to exclude the gamma band to increase consistency between systems. Future work is necessary to demonstrate that other functional connectivity measures and source reconstruction approaches^49^ offer the same consistency over time and recording systems.

In conclusion, in this longitudinal study using two different MEG systems, we demonstrated that source-space power spectral as well as functional connectivity results showed high within-subject reproducibility and remained stable over time in a group of healthy participants, despite differences in analysis pipelines and relatively long follow-up periods. Therefore, the use of the leakage-corrected AEC in source-space may allow future longitudinal analyses of the healthy and diseased brain using data recorded from different MEG systems.

## Supplementary Information

Supplementary Figures.
