# Supplementary material for: Longitudinal consistency of source-space spectral power and functional connectivity using different magnetoencephalography recording systems
Source: Sci Rep. 2021 Aug 11;11:16336. doi: 10.1038/s41598-021-95363-2 (PMC8357918; doi:10.1038/s41598-021-95363-2)

**Title**

Longitudinal consistency of source-space spectral power and functional connectivity using different magnetoencephalography recording systems.

**Authors**

Lennard I. Boon^1,2^*, Prejaas Tewarie^1,2^, Henk W. Berendse^1^, Cornelis J. Stam^1,2^, Arjan Hillebrand^1,2^

**Affiliations**

^1^ Amsterdam UMC, Vrije Universiteit Amsterdam, Department of Neurology, Amsterdam Neuroscience, De Boelelaan 1117, 1081 HV, Amsterdam The Netherlands

^2^ Amsterdam UMC, Vrije Universiteit Amsterdam, Department of Clinical Neurophysiology and Magnetoencephalography Center, Amsterdam Neuroscience, De Boelelaan 1117, 1081 HV, Amsterdam The Netherlands

***Correspondence**

Lennard I. Boon

Department of Neurology

Amsterdam UMC, location VUmc

PO Box 7057

1007 MB, Amsterdam, The Netherlands

[l.i.boon@amsterdamumc.nl](mailto:l.i.boon@amsterdamumc.nl)

**Supplementary Figure 1** Individual power spectra

Individual normalized power spectra for all subjects (all data) at three time points, averaged over all brain regions. As can be seen in this figure, in general, spectral results of the three time points visually aligned well.


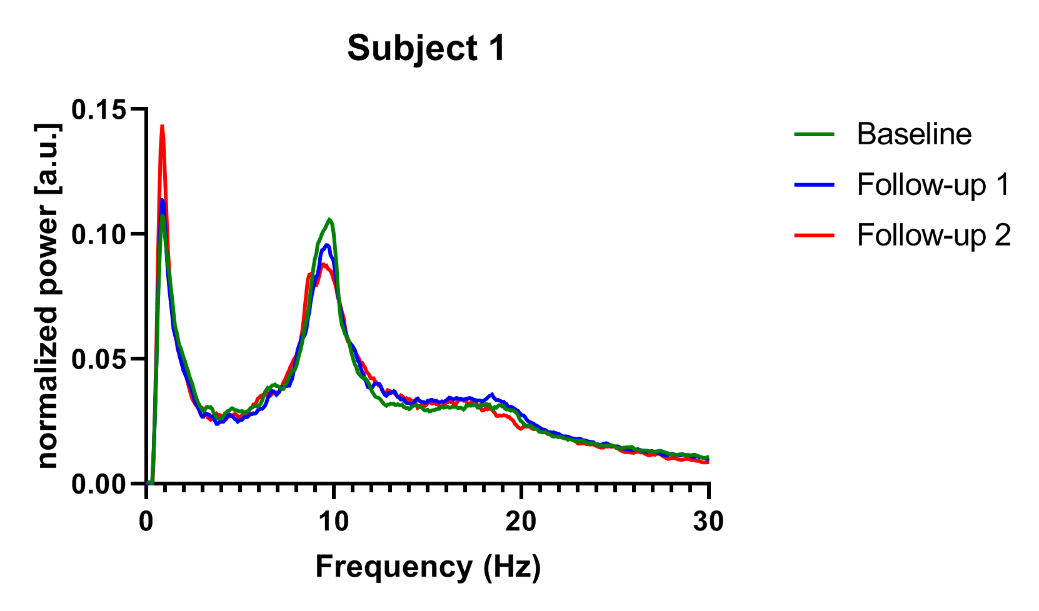


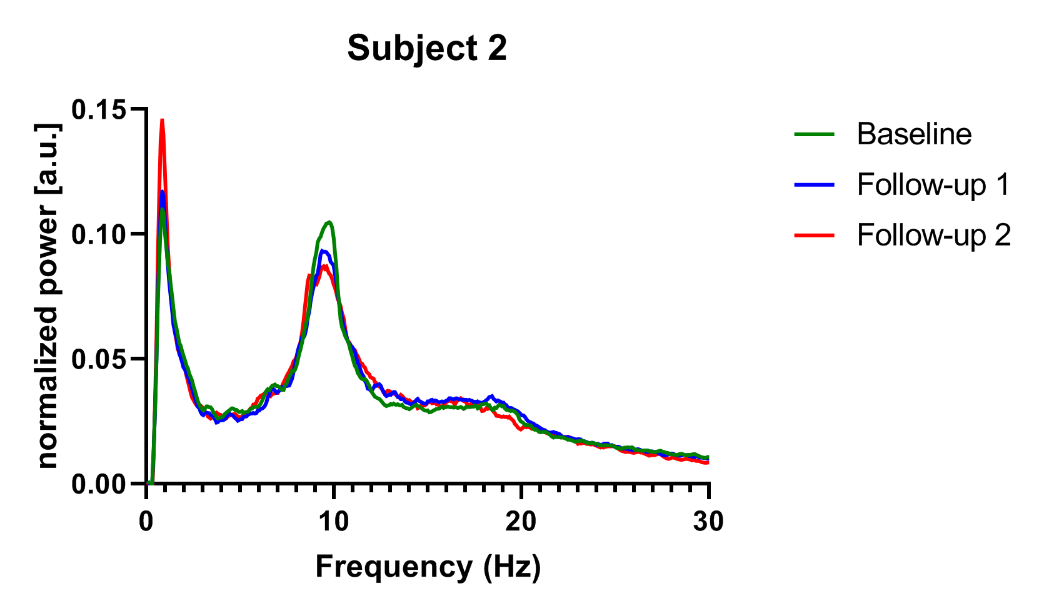


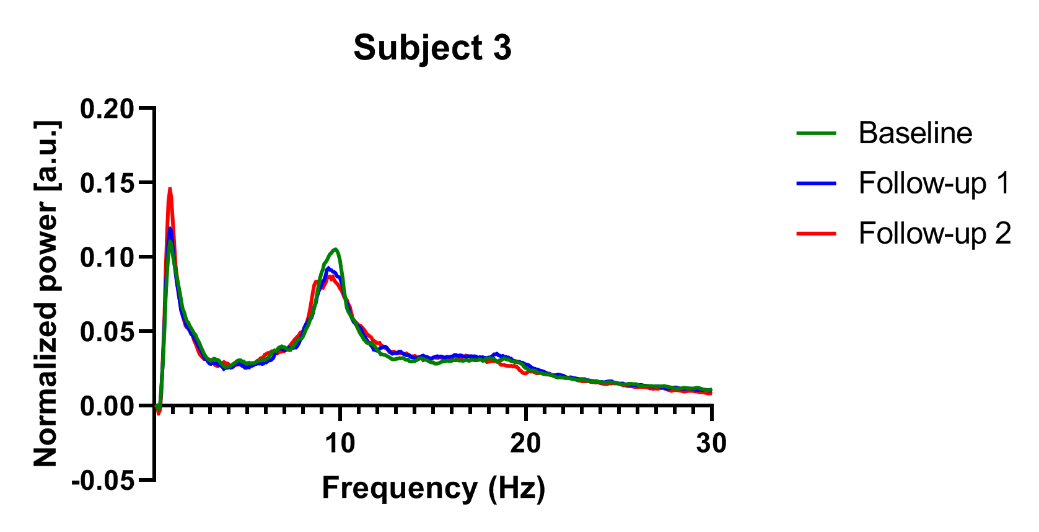


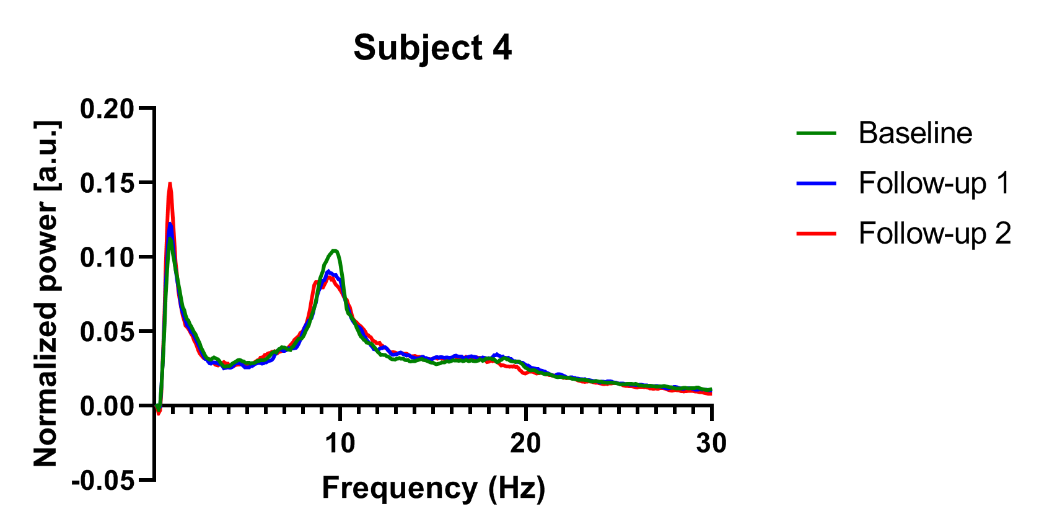


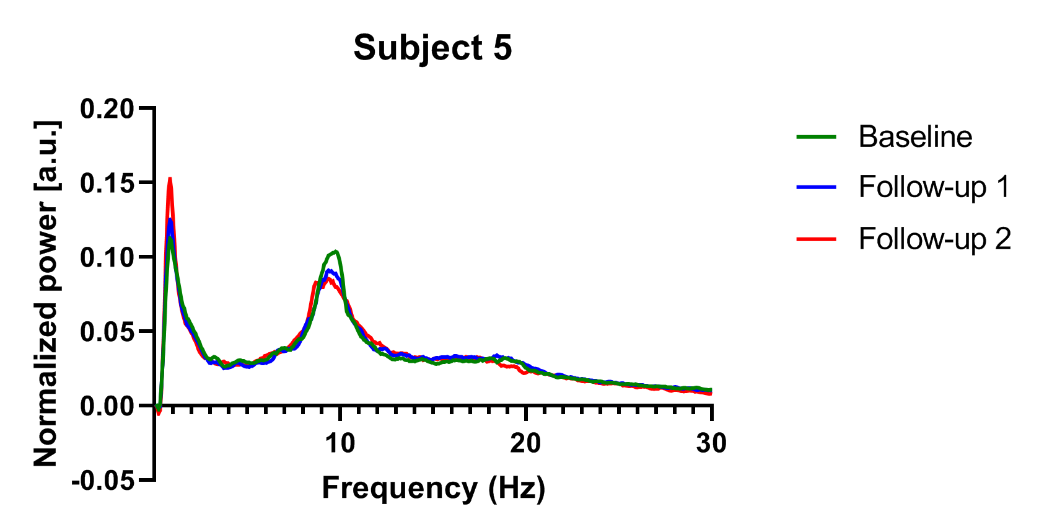


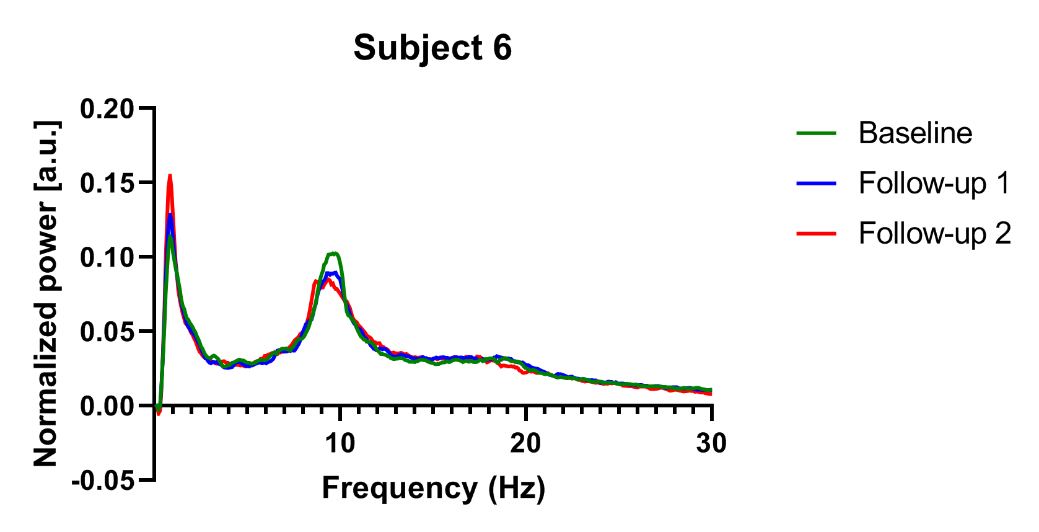

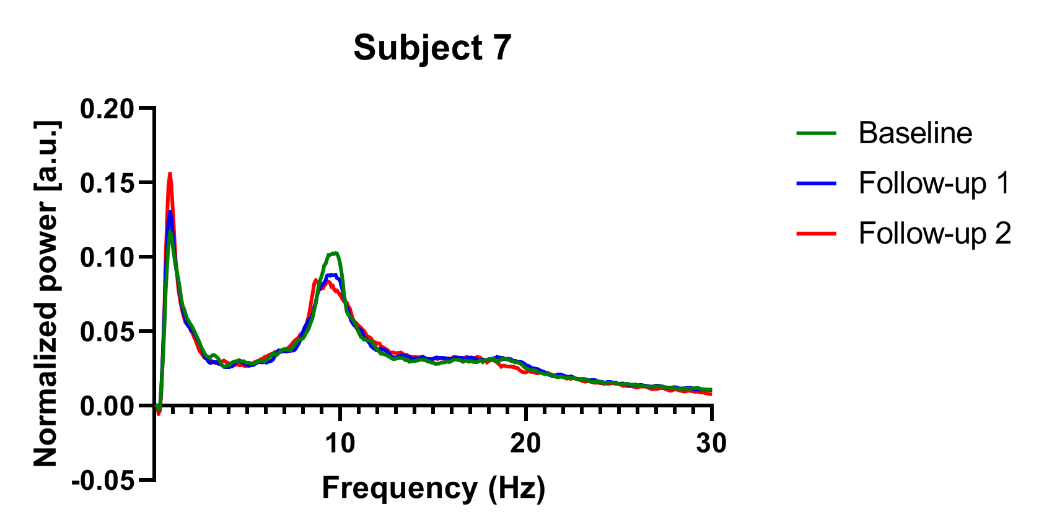

**Supplementary Figure 2**

Power spectra for averaged over all brain regions for subject 1 (FU2; 5 highest-quality epochs), both with and without tSSS. As can be substantiated from the figure, normalized power differences can be seen both in the alpha, high-beta and gamma band.


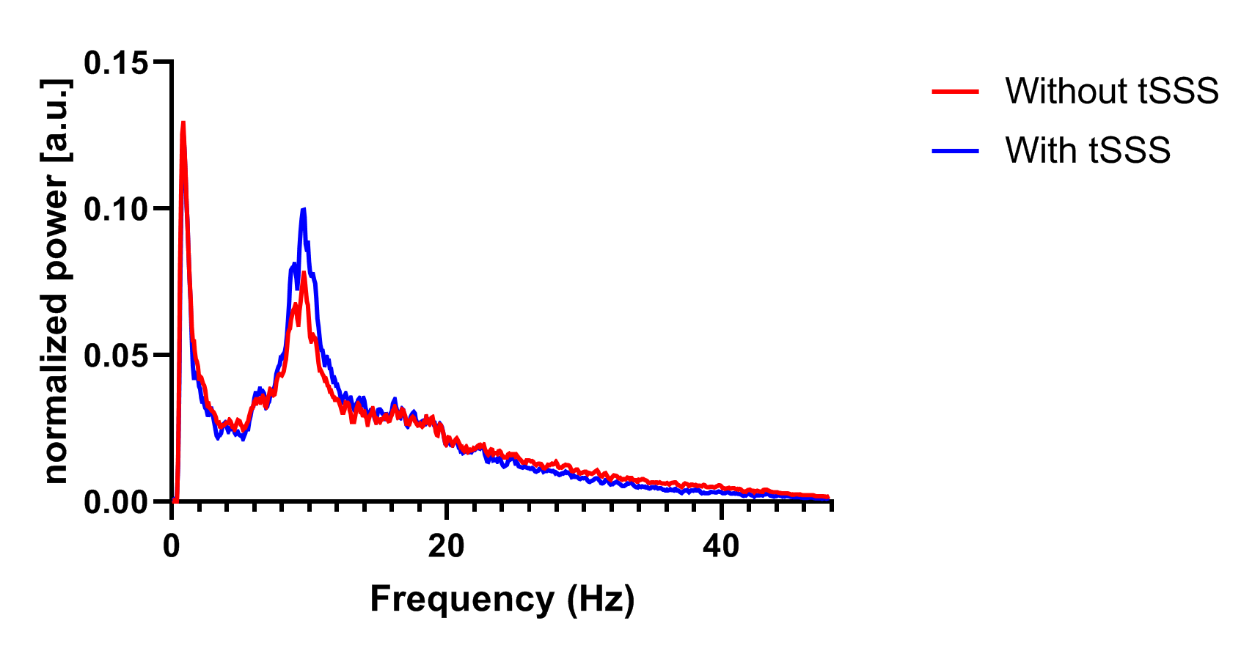

Supplement: Supplementary file 1 — Supplementary Figures. [file 41598_2021_95363_MOESM1_ESM.docx]
